# No Indication of High Host-Plant Specificity in Afrotropical Geometrid Moths

**DOI:** 10.1093/jisesa/iez028

**Published:** 2019-04-30

**Authors:** Sille Holm, Juhan Javoiš, Freerk Molleman, Robert B Davis, Erki Õunap, Heikki Roininen, Toomas Tammaru

**Affiliations:** 1 Institute of Ecology and Earth Sciences, University of Tartu, Vanemuise, Tartu, Estonia; 2 Department of Systematic Zoology, Institute of Environmental Biology, Faculty of Biology, A. Mickiewicz University, Poznań, Poland; 3 Institute of Agricultural and Environmental Sciences, Estonian University of Life Sciences, Tartu, Estonia; 4 Department of Environmental and Biological Sciences, Faculty of Science and Forestry, University of Eastern Finland, Joensuu, Finland

**Keywords:** generalist, herbivorous, latitudinal gradient, larval diet breadth, specialist

## Abstract

Specificity is one of the fundamental concepts in ecology. Host specificity of phytophagous insects has been of particular interest because of its crucial role in diversification and life-history evolution. However, the majority of tropical insects remain insufficiently explored with respect to their host-plant relations. A lack of respective data is also hindering the debate over whether higher levels of host-plant specificity prevail in tropical insects compared to temperate ones. We investigated host-plant specificity of forest geometrid moths (Lepidoptera: Geometridae) in equatorial Africa using host-plant acceptability trials with neonate larvae, with the addition of field observations. We compare our experimental data to the (well-known) host-specificity patterns of closely related temperate (hemiboreal) species. Similarly to the temperate region, there were broadly polyphagous tropical species in several clades of Geometridae utilizing hosts belonging to different plant families. Phylogenetic comparative analysis returned no significant differences in host specificity between the two regions. Our study contributes to the evidence that host-plant specificity of herbivores is not necessarily substantially higher in tropical than temperate regions.

## INTRODUCTION

Specificity (= the degree of ecological specialization, Poisot et al. 2011) is one of the fundamental parameters applied to quantify ecological interactions of a species. The causes and consequences of the variation in the degree of ecological specialization across species are at the very heart of ecological research (Futuyma and Moreno 1988, Poisot et al. 2011, Forister et al. 2012). Host-plant specificity of phytophagous insects, comprising a large proportion of animal diversity, is of particular interest as a model system of ecological specialization (Jaenike 1990, Funk et al. 2002, Forister et al. 2012).

How and why host-plant specificity varies across latitudes is a frequently asked question, but has not yet been unequivocally answered (Moles and Ollerton 2016). Although deducing general patterns from results on specific taxa must be considered with appropriate caution (Fiedler 1995, Novotny et al. 2006), taxon-specific studies constitute the primary input when addressing general patterns. Higher larval host-plant specificity in the tropics (compared to temperate areas) has been reported for Lepidoptera by Novotny et al. (2002a) and Bodner (2010). In contrast, no support for a higher degree of specificity at lower latitudes has been found for Coleoptera (Beaver 1979), Hemiptera (Hardy et al. 2016), or in some other works on Lepidoptera (Beck et al. 2006, Holm et al. 2018). Results also remain contradictory in large-scale comparisons across multiple taxa (Barone 1998, Novotny et al. 2002b, Dyer et al. 2007, Forister et al. 2015).

Furthermore, the debate on the causes of a possible latitudinal gradient in host-plant specificity is still ongoing. Plant chemistry has been considered a major determinant of specialization in phytophagous insects (Ehrlich and Raven 1964, Becerra 1997, Bernays 2001). It is generally assumed that tropical plants rely heavily on qualitative defenses such as species-specific defensive alkaloids, tannins, proteins, peptides, isoflavones, triterpenoids, and latex, with several studies showing more toxic chemicals and/or higher concentrations of these chemicals in tropical plants (reviewed in Schemske et al. 2009, Becerra 2015). Since such defenses vary among plant species, simultaneous adaptation of a herbivore to a high number of host-plant species may be complicated (Ehrlich and Raven 1964, Becerra 2015), which should lead to higher host-plant specificity in the tropics. However, other considerations would allow us to predict the opposite. For example, the diverse vegetation in the tropics increases the challenge of finding suitable plants, which should select for low host specificity. This is especially true when adults are short-lived or have little control over their dispersal (Dixon et al. 1987). Furthermore, differences in predator or parasitoid communities between temperate and tropical forests can also affect host-plant specificity (Singer and Stireman 2005, Sam et al. 2015). The effects of the third trophic level on host-plant specificity can be mediated by the tendency of specialists to have better background matching (Singer et al. 2014), or by sequestration of plant defensive chemicals by these herbivores (Nishida 2002, Singer and Stireman 2003).

Answering questions about geographic differences in host-plant specificity requires comparable data from different regions; here, however, observational data are prone to several potential sources of systematic error. First, gaps in field data (missing host-plant records) are especially likely for species from less well-studied regions such as the tropics. Tropical species are thus more likely to be erroneously considered highly specialized based on the host-plant records available (Menken et al. 2010). Second, for more polyphagous feeders, the number of host-plant species is likely to be underestimated, as some of the numerous hosts may remain unrecorded. Lastly, it is relatively harder to identify host-plant relationships that occur high in the canopy, or in other habitats that are difficult to access by researchers. Experimental approaches can avoid such problems. These include trials on oviposition and larval feeding, in which either choice (multiple-substrate trials) or acceptance (single-substrate trials; Singer 1986) can be recorded.

Geographically uneven knowledge about host-plant relationships is certainly the case for Lepidoptera, which, with about 160,000 described species (Zhang 2013), represent the second-largest radiation of phytophagous insects after Coleoptera and exhibit a bewildering variation of adaptations to detect, select, ingest, and digest live plant tissue (Menken et al. 2010). For temperate region Lepidoptera, comprehensive lists of host-plant genera are readily available (e.g., Seppänen 1970, Crafer 2005), whereas in the tropics, host relations have remained largely unknown, even for well-sampled taxa (Fiedler 1998). Missing data on host associations from the tropical regions is not only a problem in terms of understanding species’ ecology and conservation practices, but also forms a great obstacle for large-scale quantitative analyses, such as investigating patterns of specialization along a latitudinal gradient.

In the present study, we experimentally estimate larval host-plant specificity of 62 species of tropical geometrid moths (Lepidoptera: Geometridae). Geometridae is a species-rich family with a worldwide distribution, and approximately 24,000 described species (van Nieukerken et al. 2011). The typically folivorous larvae of Geometridae are often bound to woody forest plants (Scoble 1999, Hausmann 2001, Sihvonen and Skou 2015) with large among-species variation in host-plant specificity. From a technical perspective, one advantage of geometrid moths is that they easy to handle in the laboratory. In our host-plant acceptability trials, newly hatched larvae were offered the leaves of 15 tree species that are locally abundant at the study site. We then compared the results of our experiments with literature data on related temperate species, supplemented by some original results from host-plant acceptability trials. Tropical and temperate moths were compared using a phylogenetic analysis of variance (ANOVA). We also report our field observations on host-plant use of tropical geometrids. We interpret our results in the context of host-plant use of tropical insects and relate them to the debate on latitudinal gradients in specificity.

## Materials and Methods

### Larval Diet Breadth Data

#### Tropical Data

**Fig. 1.**
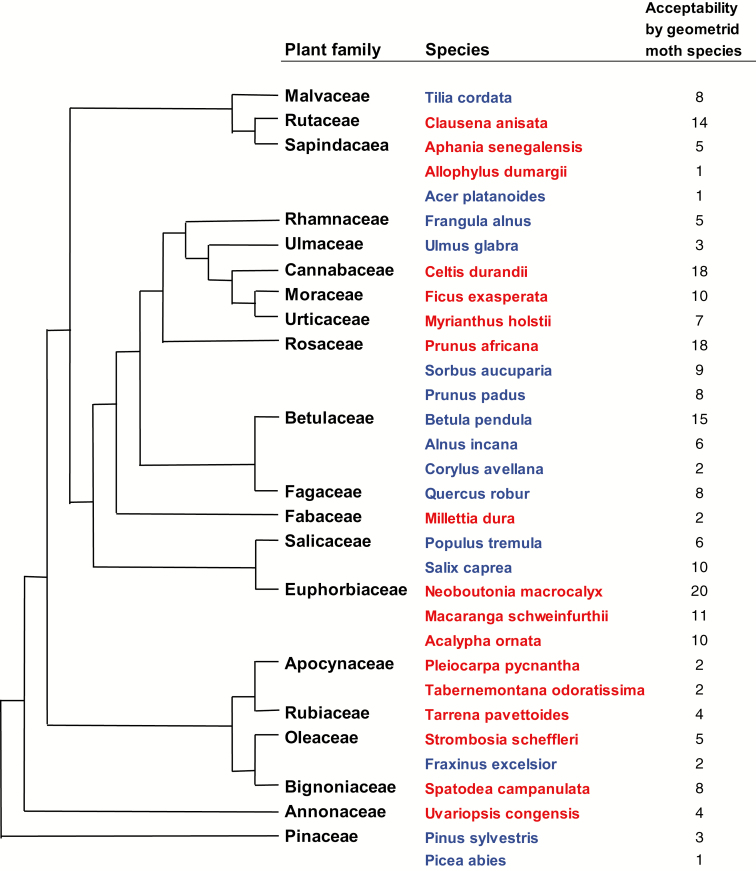
Cladogram of the plant families (modified from Song et al. 2016) and species used in this study. Species from the tropical region are marked in red, species from the temperate region are in blue.

Larval host-plant specificity of tropical geometrid moths was studied in Kibale National Park (01°N, 30°E), Uganda, East Africa. This 795 km^2^ park comprises species-rich, medium-altitude, moist, evergreen tropical forest (Struhsaker 1997). We obtained a sample from the local species assemblage of geometrids (in practice, determined by trapping success), and tested the feeding performance of the larvae on the most abundant tree species in the forest. The set of plant species considered most abundant was composed with the help of local experts, who had ample fieldwork experience in the park. The 15 tree/shrub species used in acceptance trials represent a wide array of plant families (Fig. 1), and they make up a relatively high proportion of woody plants in the local assemblage. There are no data available on their cumulative proportion at our study site but, as an educated estimate, we can expect these species to make up to 25% of all the woody plants. Seven of these species were also among the 26 species reported as being the most common for the whole national park in a study by Mucunguzi et al. (2007). The identities of plant species were subsequently confirmed by an expert at the University of Tartu. Two plant species, *Tabernamontana odoratissima* and *Allophylus dumargii*, were excluded from the experiment after the first fieldwork season and replaced with *Pleiocarpa pycnantha* and *Acalypha ornata*, because we found the former species difficult to identify in the field. Due to this change in the protocol, larvae of some moth species were offered 17 plants in total, not 15.

To obtain caterpillars for the acceptance trials, adult female geometrid moths were collected non-selectively by light trapping (years 2011–2013), and allowed to oviposit in plastic vials (see Holm et al. 2018, for details). Eggs were allowed to hatch in the same vial where laid. On the day of hatching, the caterpillars of each brood (= offspring of an individual female) were divided between the 15 plant species. If the number of hatched larvae per brood was not sufficient to cover all the 15 plant species, a random subsample of the plants was used. A fine paintbrush was used to relocate caterpillars from their native vial to the experimental arena (a 50-ml vial with a leaf of one of the 15 plant species). Three caterpillars representing the same brood were placed in each vial. Relying on more than one larva in each replicate was chosen as a way to compensate for potential losses caused by the relocation of these fragile freshly hatched larvae. After 48 h, the response of the larvae to the plant was recorded: 0 – plant not accepted: no feeding marks on the leaf, no feces; 1 – accepted: obvious signs of consumption, abundant feces, caterpillars have grown larger. The few unclear cases were omitted from the analysis.

Acceptance of a host plant by a neonate larvae does not necessarily imply that the insect is able to develop on the plant to adulthood. We, therefore, complemented our data set by indicating the cases when we reared a larva on a certain plant to the adult stage. The larvae were reared to adults in the context of another (sub)project (Holm et al. in review), and such rearing attempts were performed for only a minority of moth species × host-plant species combinations examined in the present paper. We, therefore, abstain from including these data in a formal analysis but report them along with our experimental results (Table 1). For the geometrid species included in the present study, we also report host plant records we made in the field in Kibale between 2011 and 2013 (Table 1).

**Table 1.**
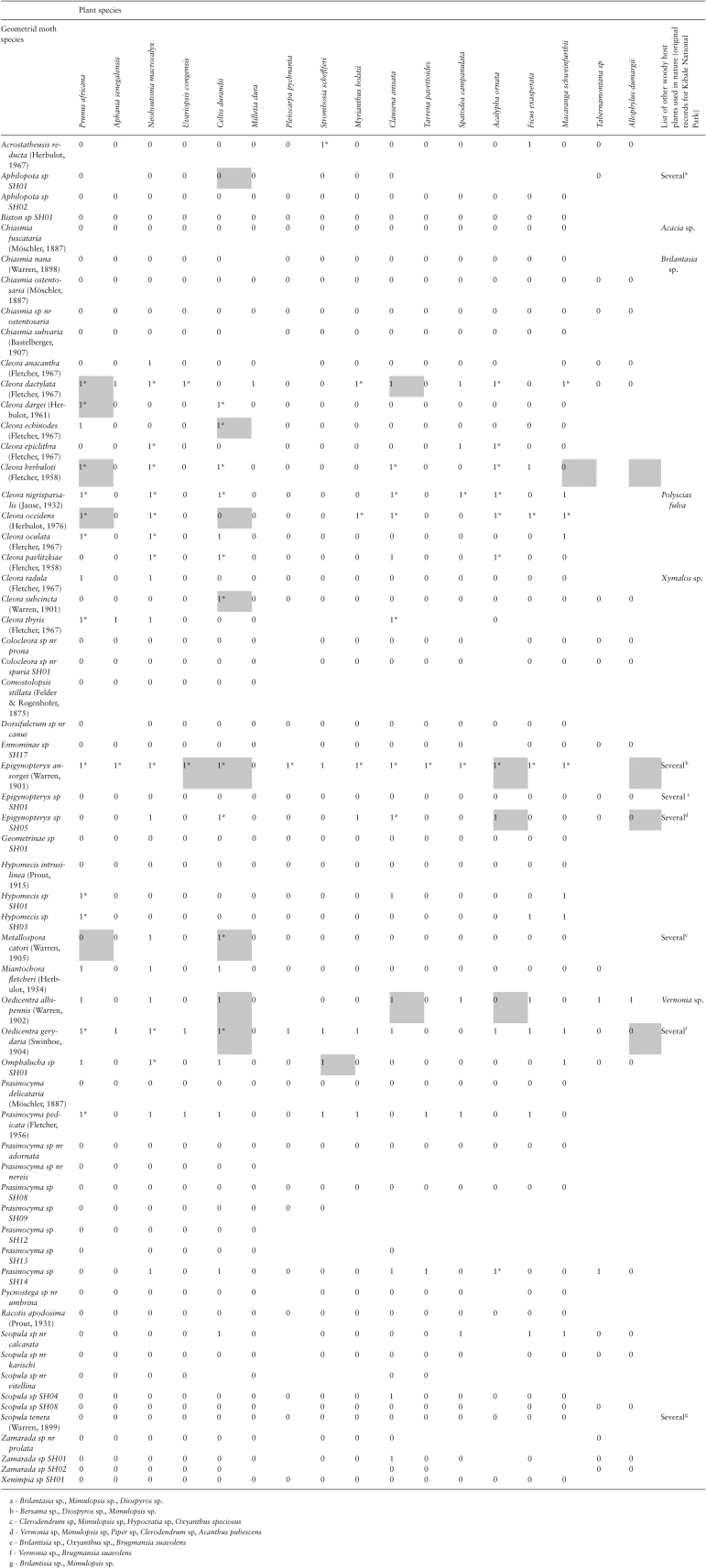
Results of host-plant acceptability trials with neonate larvae of Ugandan Geometridae. Positive results – ‘plant accepted’ – are coded as 1s in the table (see Material and Methods for details). The data are supplemented with records of successful development on particular host plants to the adult stage (original observations of the authors; indicated with an asterisk (*)), and original host-plant records from the field (indicated with grey background).

#### Tropical Moth Species Identification

Tropical specimens were brought post hoc to Estonia to be identified, and are stored at the facilities of the Department of Zoology, University of Tartu. Species identification was based on external traits (coll. Herbulot at the Zoological Museum of Munich was consulted) or genitalia dissection, and species’ DNA barcodes were cross-checked against the DNA barcodes in the Barcode of Life Data System (http://www.boldsystems.org/index.php/IDS_OpenIdEngine) (Ratnasingham and Hebert 2007). Nevertheless, some moths could not be reliably assigned to any named species; these taxa were instead given biospecies names (e.g., *Epigynopteryx_sp_SH01*) for the analysis. Any unidentified individuals were considered conspecific if there was less than a 2% difference in barcode sequences.

#### Temperate Region Data

To compare larval host-plant specificity of geometrid moth species from tropical and temperate zones, we identified the closest relatives (conditional sister groups) of the species studied in Uganda that inhabit the hemiboreal zone of Northern Europe (approximately, between the northern latitudes of 57° and 61°). For identification of the conditional sister groups an original phylogeny was used (see section below). The sample of temperate species considered (157 spp.) was based on that used in our previous phylogenetic comparative works (Davis et al. 2016; Holm et al. 2016, 2018). Our temperate sample (though not complete) covers the north-European fauna well enough so it is likely that the closest relatives of the studied tropical moths are correctly identified at least to the genus level.

In 2018, we performed host-plant acceptability trials with six hemiboreal species belonging to the temperate sister groups of the studied Ugandan species. The trials followed the same protocol as described above for the tropical region. To provide a selection of host-plant species comparable to that used in the tropics, we chose the 15 most widely distributed and common forest tree species (Table 2) in the hemiboreal region, using Estonia as a reference location (Leemet and Karoles 1995, personal observation of the authors). As the tropical sample did not include any congenerics, the same principle was applied for the list of temperate plant species. The host-plant acceptability experiments with neonate larvae of temperate species were performed in Estonia in June and July 2018.

**Table 2.**
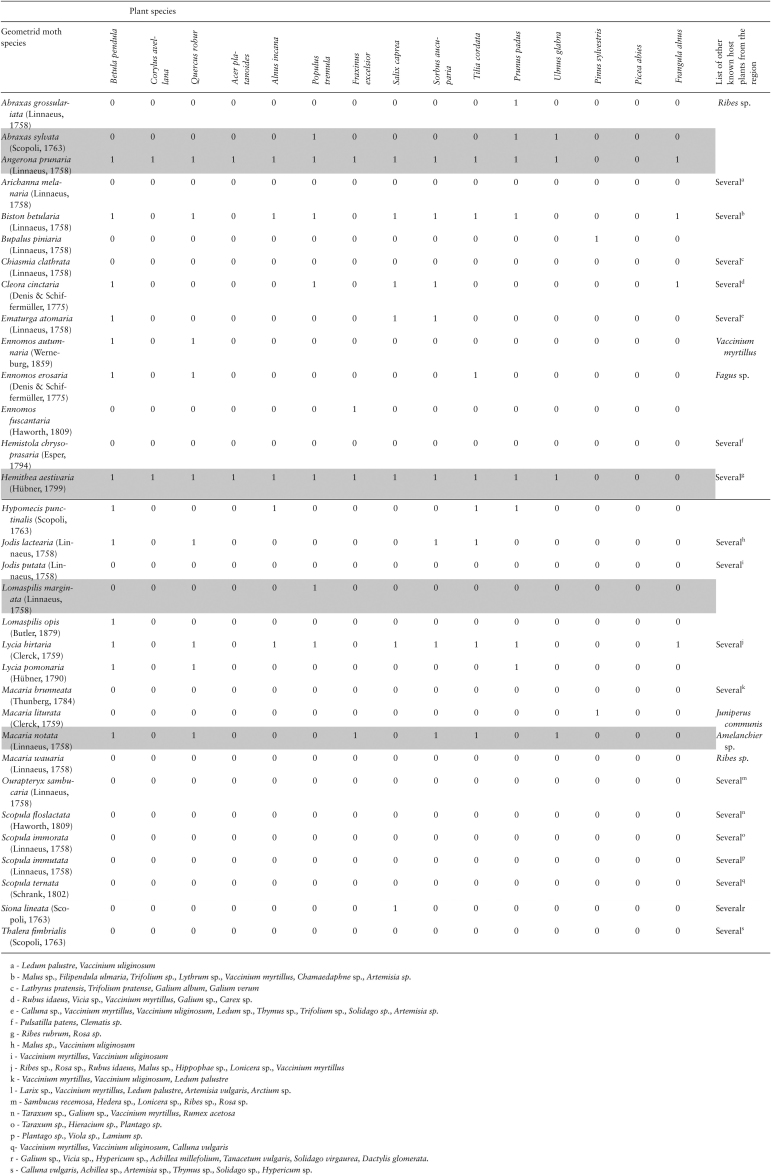
Acceptability (1 – acceptable, 0 – not acceptable as a host plant) of the 15 most common tree species by geometrid moth species in hemiboreal Europe. Data retrieved from literature are indicated with a white background; data derived from acceptability trials with neonate larvae are indicated with a grey background (see Material and Methods for details).

For the rest of the temperate species, we retrieved literature-based data on diet breadth. Scoring was based on host-plant lists published in handbooks (Seppänen 1970; Mikkola et al. 1985, 1989) on the moth fauna of Finland, a country adjacent to Estonia. We used data reported in Leraut (2009) for some species (*Hemithea aestivaria* [Hübner, 1799]; *Hemistola chrysoprasaria* [Esper, 1794]) whose data were not available in the Finnish handbooks. In general, however, using host-plant data from more distant regions was avoided to keep the temperate data as comparable as possible to the tropical data set, which was itself based on just one location.

Naturally, despite good knowledge of the ecology of north-European moths, such literature data need not be quantitatively comparable with the data derived from our experiments. It appears likely that the literature adequately reflects the host-plant specificity of mono- and oligophagous moths, due to the amateur naturalist’s interest in establishing host relationships of such species. Nevertheless, we suspect that handbook-retrieved data underestimate the list of acceptable host plants for polyphages (broad generalists), primarily because of naturalists’ (primary providers of handbook data) limited motivation in recording and reporting the use of particular host plants by moth species known to be polyphagous.

Our host-plant acceptability experiment with hemiboreal species, indeed, confirmed the underestimation of the host range in polyphagous species. For instance, based on literature sources, we would have characterized *Angerona prunaria* (Linnaeus, 1758) as using four tree species out of the 15 offered, whereas our experiment returned an acceptance of 13 host-plant species. To address the problem of possible underestimation of the host use in literature-based data for polyphagous species, we performed alternative analyses on the data set in which all moth species characterized as ‘polyphagous on deciduous trees’ by Mikkola et al. (1985, 1989) were rescored as accepting all 13 deciduous trees in our list of 15 host-plant species.

#### Phylogenetic Tree

The phylogenetic tree of geometrid moths was constructed on the basis of both data submitted to GenBank by earlier researchers (Wahlberg et al. 2005, 2010; Snäll et al. 2007; Viidalepp et al. 2007; Õunap et al. 2008, 2011, 2016; Wahlberg et al. 2013; Mutanen et al. 2010; Strutzenberger et al. 2010; Hausmann et al. 2011; Sihvonen et al. 2011; Huemer et al. 2014; Holm et al. 2016, 2018; Tammaru et al. 2018; Zrzavá et al. 2018) and original sequences of both Ugandan and Estonian species, which were obtained following reaction protocols described in Õunap et al. (2016). The final data matrix comprised 373 taxa and 6543 base pairs from eight markers that have repeatedly been used for phylogenetic inference in geometrid moths. All sequences were aligned using CLUSTALW (Thompson et al. 1994) in BIOEDIT 7.2.5 (Hall 1999) and analyzed by using BEAST 1.8.1. (Drummond et al. 2012). See Supp. Appendix 1 (Gene data used to conduct Geometridae phylogeny); Supp. Appendix 2 (Construction of the phylogeny of Geometridae); Supp. Appendix 3 (Geometridae phylogeny), for more details.

### Data Analysis

**Fig. 2.**
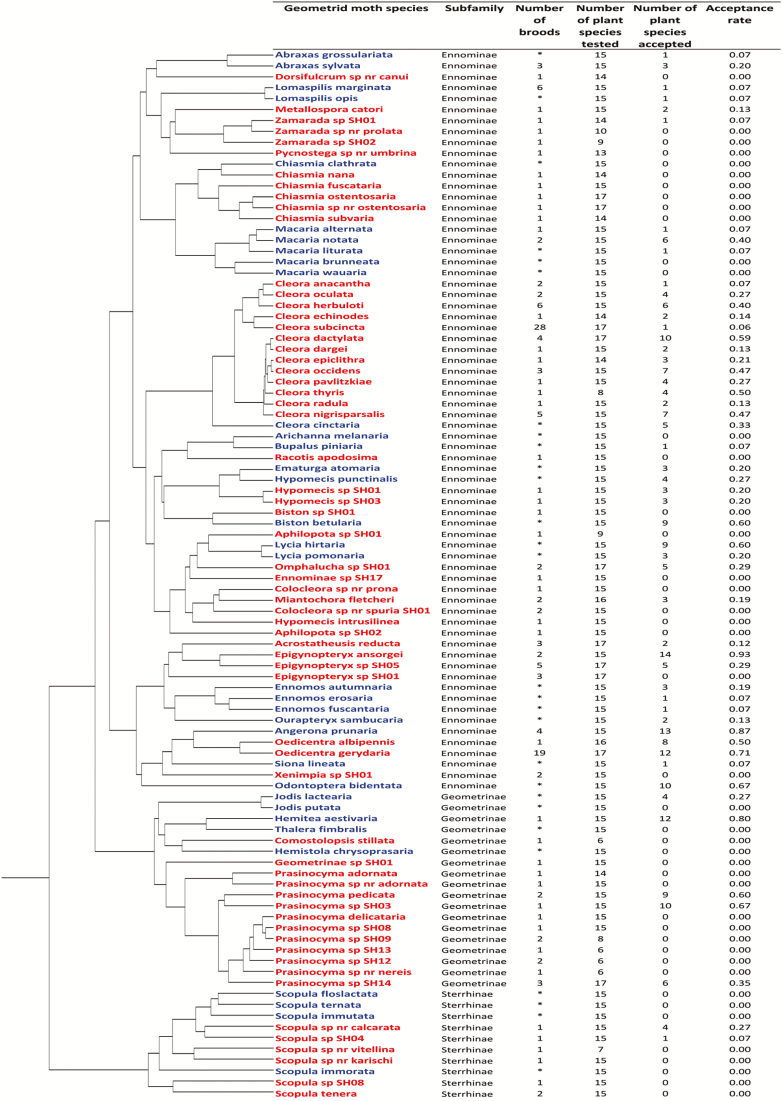
Phylogeny of Geometridae used in this study with details on sample sizes and acceptance rate of the host plants offered. Data for the tropical region (marked in red) were obtained in the acceptability trials in Uganda. Data for the temperate region (marked in blue) are based on literature (*) on hemiboreal species with the exception of *Abraxas sylvata, Angerona prunaria, Hemithea aestivaria, Lomaspilis marginata, Macaria alternata, and M. notata,* for which data was obtained in acceptability trials.

In order to obtain an index of host-plant specificity for each moth species (both tropical and temperate), we calculated the percentage of plant species accepted by the larvae (number of plant species accepted divided by number of plant species offered; Tables 1 and 2; Fig. 2); referred to as *acceptance rate* hereafter. In the case of within-species (among-brood) variation in acceptance patterns, one positive result (acceptance) was considered sufficient to score the species as accepting the plant in question. Such an approach was chosen to make the empirical data comparable to the literature data on temperate species in which inclusion of a tree species on a host-plant list does not imply that all individuals of the moth species are able to utilize it. Unfortunately, our data were not sufficient for a meaningful analysis of within-species variation in host-plant use.

To study the differences in acceptance rate between regions (temperate vs tropical) in a phylogenetic context, we conducted a phylogenetic ANOVA, using the function phylANOVA in the package phytools (Revell 2012) for the R statistical environment (R Development Core Team 2012). This function performs a simulation-based phylogenetic ANOVA based on Garland et al. (1993) with posthoc tests comparing the group means. We used data obtained from host-plant acceptability trials for species for which we had such data available.

## Results

### Tropical Region

In the host-plant acceptability trials performed in the tropical region (Kibale National Park in Uganda), we scored the larval diet breadth of 62 geometrid moth species. In total, 614 female geometrids were collected via light trapping: 297 of those laid eggs, 190 broods hatched, and 142 of these were identified to the species level. Only three species that had a sample size of more than two individuals per species completely failed to oviposit in our laboratory conditions. The average number of broods per species was 2.3 with a maximum of 28 for *Cleora subcincta* (Warren, 1901). The average number of plant species offered in one trial was 14 (i.e., slightly fewer than the intended 15 as the number of larvae was sometimes limiting). A positive result (‘acceptance’) was obtained for 141 out of 868 moth species × plant species combinations (16%). The highest acceptance rate was 0.93 (14/15) for the moth *Epigynopteryx ansorgei* (Warren, 1901), followed by *Oedicentra gerydaria* (Swinhoe,1904) with 0.70 (12/17). Twenty-five moth species accepted two or more plant species—different plant families were involved in all such cases (Fig. 1). Four moth species accepted only one of the plants offered, whereas the neonate larvae of 33 moth species did not accept any of the plants (Table 1; Fig. 2). There was considerable between-species variation in host-plant acceptance rate even among congeneric moths; the moths from the genus *Cleora* (Curtis, 1825) (represented by 13 species) showed different acceptance patterns both within individual plant families (e.g., Euphorbiaceae represented by three species) and between plant families (Table 1; Fig. 1), indicating weak phylogenetic constraints on host-plant specificity.

Rearing the insects to the adult stage was not systematically attempted in the framework of the present project. Nevertheless, we have evidence that many of the tropical species successfully completed their development on various tree species representing the local flora, and were found to use different plants in the field (Table 1). These observations support the reliability of the host-plant acceptance trials with neonate larvae as a source of information on host-plant use, and contribute to conclusions about the potentially broad host-plant spectrum of many Ugandan forest geometrids.

### Temperate Region

Of the six hemiboreal species we had experimental data for (Table 2; Fig. 2), two were found to be broad generalists. The experimentally derived host-plant acceptance rate for *A. prunaria* of 0.87 (13/15) was the highest score within the temperate set of species. The remaining four species were found to be oligophagous (Table 2), which is consistent with literature data (Mikkola 1985, 1989).

The highest literature-based estimate of acceptance rate was 0.67 (*Odontopera bidentata* (Clerck, 1759), 10 plants out of 15; Fig. 2). Eleven out of the 34 temperate region moth species considered (32%) were not known to accept any of the plant species from our list of 15 trees/shrubs (Table 2; Fig. 2); these are known to feed on either herbaceous plants, dwarf shrubs or tree species not on our list.

### Comparison Between Regions

We found no significant effect of region on host-plant specificity (Phylogenentic ANOVA: *F* = 0.554, *P* = 0.58, with ‘acceptance rate’ as the response variable and ‘region’ as a fixed factor). Overall, the temperate moths included in the analysis were found to accept just a slightly higher rate of the plants on our list, compared to the tropical species (0.19 for the temperate and 0.15 for the tropical region, on average).

The analysis of the total data set (Fig. 2) suffers from a problem with ‘zeroes’. A species that did not accept any of the offered tree species may not be a specialized tree feeder, but may be a herb feeder instead, perhaps even a generalist herb feeder. We were able to identify such cases for the temperate moths (host-plant specificity known) but not for representatives of the tropical fauna. In an attempt to address this problem, we performed an alternative analysis in which we omitted all species with zero acceptance rate from both regional samples (23 for the temperate region; 29 for the tropical region). This way we left only confirmed tree feeders in our sample, at the risk of omitting some specialized tree feeders (feeding on both trees and herbs is rare among Geometridae; Mikkola et al. 1985, 1989). The average acceptance rate shifted towards a higher value for the tropical region (0.28 for the temperate vs 0.32 for the tropical region), with the difference remaining nonsignificant (*F* = 0.224, *P* = 0.79).

In a second alternative analysis, we addressed the problem of the likely underestimation of diet breadth in the literature-based data for polyphagous temperate species. We modified our data set so that the seven species reported to be polyphagous species were scored as accepting all deciduous trees. Naturally, the among-region difference in average acceptance rate shifted towards a higher value (0.27 for the temperate vs 0.15 for the tropical region), but still remained nonsignificant (*F* = 4.07, *P* = 0.18). The among-region difference was even smaller when, in a third alternative analysis, we removed all species that did not accept any of the offered tree species from our data set (0.40 for the temperate vs 0.32 for the tropical region; *F* = 0.860, *P* = 0.6).

## Discussion

Comparing host-plant acceptance rates between temperate north-European and equatorial east-African geometrid moths, we found qualitative patterns of host-plant specificity to be similar. We did not thus find support for the hypothesis that tropical species have higher specificity than temperate ones. Average host-plant acceptance rate was found to differ only moderately between the regions, with no statistical significance attained in any of the alternative phylogenetically informed analyses. Even if results of the quantitative analyses are to be treated with caution (see below), some qualitative patterns are clearly robust and may alone serve as sufficient basis for our primary conclusions. In particular, among the Geometridae of both the studied tropical region and the temperate region chosen for comparison, there are species that are able to feed on the clear majority of common tree species in the forest. In both regions, such polyphagous moths are found in several distantly related clades of Geometridae. Moreover, moth species that are able to use forest trees from different families appear to constitute a substantial proportion of the geometrid fauna in both regions, so that diet breadths appear to be similar across regions also in terms of phylogenetic diversity of host plants used (Fig. 1). This shows that despite the presumably higher levels of chemical defense in tropical trees (Schemske et al. 2009), geometrid moths are still able to exploit a large variety of tree species in an equatorial African forest. The qualitative similarity in host-plant specificity between the species assemblages inhabiting the two latitudinally distant study sites thus does not accord with recent global-scale studies on Lepidoptera (Dyer et al. 2007, Forister et al. 2015) that report a substantial increase in specificity towards the tropics. In particular, Forister et al. (2015) found that at sites above a 25° latitude 60% of caterpillar species are associated with a single host family, and at sites below a 25° latitude this value is 83%. Quite interestingly, our conservative estimate is that just 60% of the species were associated with single host family at the studied 01°N latitude site. We suppose that the inconsistency with previous results is primarily associated with differences among lepidopteran taxa considered.

While we see no reason to question the overall pattern of host-plant specificity detected for the equatorial African Geometridae, we would still like to discuss some methodological aspects which may interfere with the quantitative results of the study. First, our host-plant acceptability trials were based on the premise that most of the species caught in the forest habitat in Uganda are tree feeders. Indeed, feeding on woody plants is characteristic of the family (Scoble 1999), especially in the tropics (Holloway 1996), and very few species of Geometridae are able to feed on both trees and herbs (Mikkola et al. 1985, 1989). In fact, however, the number of herb feeders among the tropical sample of species is not known. Despite this, an alternative analysis conservatively excluding all species which did not accept any of the offered tree species did not lead to any different conclusions. Thus, our result appears robust concerning this issue. Second, as a consequence of our study design, our tropical study sample was biased towards the most common and abundant moth species at the site. It is, however, possible that the common species may be less specialized than random representatives of the assemblage. If true, this would mean an underestimation of host-plant specificity in the Ugandan fauna. This problem is nevertheless mitigated by the fact that most temperate species used for the comparison are abundant in their habitats as well. The species sets studied are comparable between the regions because roughly 10% of the total geometrid fauna was covered in both sites. A third aspect is that we may have missed some of the more specialized Ugandan species simply because specialists are less likely to oviposit in vials with no host plant present (Holm et al. 2018); however, there were just three species which failed to oviposit in captivity (see Results). Fourth, some uncertainty inevitably remains with respect to quantitative comparability of literature data and our experimental results. However, because our two approaches to scoring host-plant specificity in the temperate sample (one likely underestimating, the other overestimating the number of hosts of the less specialized species) did not lead to qualitatively different results, we are convinced that the lack of a qualitative difference between the sites is robust.

Finally, it should be noted that our host-plant acceptance trials measured potential host range of the studied species (what they are able to eat), whereas the realized host range of a herbivorous insect (what they actually do eat) can be affected by factors other than the biochemical composition of plant leaves (Bernays and Chapman 1994). Nevertheless, we have reasons to assume that, in Geometridae, the difference between potential and realized host range is not so dramatic. This is because geometrid females are not particularly selective with respect to oviposition site (in the Ugandan sample as well, most females readily laid eggs without a host plant present, Holm et al. 2018). Moreover, the larvae of the folivorous species are mobile enough to move between host plants (e.g., being carried around between trees by the wind when ballooning as small larvae, and climbing up trees when they fall down from a tree as large larvae, e.g., Tammaru et al. 1995), so we assume that they can be found feeding on any plants they are able to consume. Moreover, the idea about good consistency between the potential and the realized niche is directly supported by our host-plant records from the field (Table 1).

A relatively high rate of polyphagy in tropical Geometridae has recently been suggested by field studies in different regions (e.g., Brehm 2002, 2003). Similarly, we have shown that oviposition behavior of geometrid moths is not indicative of a higher degree of host-plant specificity in this tropical forest than in Northern Europe (Holm et al. 2018), corroborating the results of the current study. The possibility that the Geometridae, therefore, represent a taxon that does not follow the frequently proposed latitudinal trend of increasing specificity towards the equator thus deserves attention (see Hardy et al. 2016 for similar examples).

Specialization is generally considered possible when the availability of the host resource is stable (Beaver 1979, but see Põldmaa et al. 2016). Due to high plant diversity in the tropics, high risk of predation (Roslin et al. 2017) and the rather short lifespans of geometrid moths (Holm et al. 2016, 2018), locating preferred host plants among the myriad of chemical cues in the environment may be a challenge for a specialist. Also, compared to many other Lepidoptera, the adults of geometrids are known to be of rather limited mobility , especially those capital-breeding females that carry large egg loads in their abdomens (Snäll et al. 2007, Davis et al. 2016). Limited mobility adds another challenge to locating a suitable host plant (or plants), and temperate geometrids thus frequently rely on completely indiscriminate oviposition, with larval dispersal playing an important role in host finding instead (Tammaru et al. 1995). Careful species-specific studies on tropical geometrids are needed to discern if the behavioral patterns are similar in the tropics as well. At a more general level, such studies will be useful to confirm or refute the frequent belief that the environments encountered by temperate and tropical insects impose considerably different selection pressures.

In conclusion, as long as the biodiversity of tropical habitats continues to be insufficiently studied, many questions regarding the degree of specificity of tropical species will remain without satisfactory answers. Our results, however, suggest that geometrid moths in Uganda are not substantially more specialized than closely related temperate species of Northern Europe. Although the results of the present study cannot be generalized to make a broad comparison across biomes, we have nevertheless contributed evidence indicating that temperate and tropical species may not be so different in their specificity. On the other hand, recorded patterns of host use indicate that host-plant specificity is perhaps not strongly constrained phylogenetically. In the future, a larger sample of geometrid species would provide the basis for a meaningful analysis of phylogenetic constraints in host-plant use, but this requires the sampling of further sites and communities along a latitudinal gradient.

## Supplementary Material

iez028_suppl_Supplementary_Appendix_1

iez028_suppl_Supplementary_Appendix_2

iez028_suppl_Supplementary_Appendix_3
