# Supplementary material for: No Indication of High Host-Plant Specificity in Afrotropical Geometrid Moths
Source: J Insect Sci. 2019 Apr 30;19(3):1. doi: 10.1093/jisesa/iez028 (PMC6490970; doi:10.1093/jisesa/iez028)
Supplement: iez028_suppl_Supplementary_Appendix_2 [file iez028_suppl_supplementary_appendix_2.docx]

**Appendix 2 Construction of the phylogeny of Geometridae**

***Molecular data***

The phylogenetic tree used for analysis in this research was constructed on the basis of both data submitted to GenBank by earlier researchers (Wahlberg *et al*., 2005, 2010; Snäll *et al*., 2007; Viidalepp *et al*., 2007; Õunap *et al*., 2008, 2011, 2016; Wahlberg & Wheat, 2008; Mutanen *et al*., 2010; Strutzenberger *et al*., 2010; Hausmann *et al*., 2011; Sihvonen *et al*., 2011; Huemer *et al.*, 2014; Holm *et al.*, 2016, 2018; Tammaru *et al.*, 2018; Zrzavá *et al.*, 2018) and original sequences, which were obtained following reaction protocols described in Õunap et al. (2016). The final data matrix comprised 373 taxa and 6543 base pairs from eight markers that have repeatedly been used for phylogenetic inference in geometrid moths: cytochrome oxidase subunit 1 (COI), elongation factor 1 alpha (EF-1a), wingless (wgl), glyceraldehyde-3-phosphate dehydrogenase (GAPDH), ribosomal protein S5 (RpS5), isocitrate dehydrogenase (IDH), malate dehydrogenase (MDH) and carbamoylphosphate synthase domain protein (CAD) (see also Appendix 1_Gene data used to conduct Geometridae phylogeny). All sequences were aligned using ClustalW (Thompson et al. 1994) in BioEdit 7.2.5 (Hall 1999).

***Phylogenetic analyses***

As the number of taxa analysed in this study exceeded 350, it proved to be impractical to analyse them all together to obtain an ultrametric tree required for subsequent statistical analysis. Therefore, two-step analysis was designed to overcome difficulties related with the size of a full data matrix. These steps are hereby described briefly to give the reader a general overview of the process, with detailed description of each step being following below.

First, four ultrametric trees were created using Beast 1.8.1. (Drummond *et al.*, 2102) on the CIPRES Science Gateway (Miller et al., 2010). All these trees shared 22 Palaearctic taxa from subfamilies Archiearinae, Larentiinae, Sterrhinae, Geometrinae and Ennominae, but were focussed on a different subset of Geometridae: the first one on Geometrinae, the second one Ennominae, the third one on Larentiinae, and the fourth one on Sterrhinae, respectively.

Second, the four ultrametric trees were combined into a single 373-species ultrametric tree (Supplementary material Appendix 3) using the consensus.edges function from the R package ‘phytools’ (Revell 2012). For the purposes of the present study, the tree was pruned based on the availability of host use data (Figure 2).

First step: constructing ultrametric trees for subfamilies

*Ennominae*

Analysis focussed on the subfamily Ennominae comprised a total of 197 species. For phylogenetic reconstruction, we first determined the best partitioning scheme from three competing scenarios. For the first scenario, each gene was defined as a separate partition; for the second scenario, each codon position (1^st^, 2^nd^ and 3^rd^) of each gene was defined as a separate partition; for the third scenario, the data were divided between 50 subsets (bins) according to their evolutionary rates using Tiger v 1.02 (Cummins and McInerney 2011). To choose between these scenarios, a ML tree following GTR+I+G model was first constructed using Mega6 (Tamura et al. 2013). This tree was used as user_tree_topology in PartitionFinder 1.1.1 (Lanfear et al. 2012) to find the best partitioning scheme for all three competing scenarios. Bayesian Information Criterion (BIC) values of the best scheme for each scenario were compared and the codon-based treatment was selected for further calculations.

According to the suggestion by PartitionFinder, nucleotide data were divided into three partitions, each comprising nucleotides corresponding to at least one codon position of at least one gene. GTR+I+G model was implemented on each partition according to suggestions by PartitionFinder. A relaxed molecular clock allowing branch lengths to vary according to uncorrelated lognormal distribution (Drummond et al. 2006) was used for each partition. To calibrate the ultrametric tree, four calibration points were defined according to Wahlberg et al. 2013: root of Geometridae (age 71.5 million years, with uniform distribution between 60.9 and 82.6 million years), split between Archiearinae and Geometrinae+Ennominae lineage (age 60.5 million years, with uniform distribution between 49 and 71.5 million years), split between Ennominae and Geometrinae (age 46.1 million years, with uniform distribution between 35.8 and 56.2 million years), and split between Sterrhinae and Larentiinae (age 56 million years, with uniform distribution between 42.3 and 69.5 million years). The tree prior was set to the Birth–Death process (Gernhard 2008) whereas the ucld.mean parameter was given a uniform prior between 0 and 1 for all partitions. All other priors were let to defaults. Seven independent analyses were performed, with Bayesian MCMC running for 20 million generations and sampling every 1000^th^ generation. The results were inspected in Tracer 1.5, and 2 million, 3.5 million, 7 million, 10.2 million, 1 million, 1 million and 5 million generations were discarded from each run as ‘burn-in’, respectively. Thereafter, the remaining results of all analyses were combined together using LogCombiner v1.6.2 (a supplementary software to Beast). The final tree was constructed using TreeAnnotator v1.6.2 (also a supplementary software to Beast) in the High Performance Computing Center of University of Tartu and visualized in FigTree v1.4.0.

*Geometrinae*

Analysis focussed on the subfamily Geometrinae comprised a total of 86 species. For phylogenetic reconstruction, we first determined the best partitioning scheme from three competing scenarios. For the first scenario, each gene was defined as a separate partition; for the second scenario, each codon position (1^st^, 2^nd^ and 3^rd^) of each gene was defined as a separate partition; for the third scenario, the data were divided between 50 subsets (bins) according to their evolutionary rates using Tiger v 1.02. To choose between these scenarios, a ML tree following GTR+I+G model was first constructed using Mega6. This tree was used as user_tree_topology in PartitionFinder 1.1.1 to find the best partitioning scheme for all three competing scenarios. Bayesian Information Criterion (BIC) values of the best scheme for each scenario were compared and the bin-based treatment was selected for further calculations.

According to the suggestion by PartitionFinder, nucleotide data were divided into four partitions, each comprising one or more bins. HKY+G and GTR+I+G models were both implemented on one, and GTR+G model on two partitions according to suggestions by PartitionFinder. A relaxed molecular clock allowing branch lengths to vary according to uncorrelated lognormal distribution was used for each partition. To calibrate the ultrametric tree, four calibration points were defined according to Wahlberg et al. 2013: root of Geometridae (age 71,5 million years, with uniform distribution between 60.9 and 82.6 million years), split between Archiearinae and Geometrinae+Ennominae lineage (age 60.5 million years, with uniform distribution between 49 and 71,5 million years), split between Ennominae and Geometrinae (age 46.1 million years, with uniform distribution between 35.8 and 56.2 million years), and split between Sterrhinae and Larentiinae (age 56 million years, with uniform distribution between 42.3 and 69.5 million years). The tree prior was set to the Birth–Death process whereas the ucld.mean parameter was given a uniform prior between 0 and 1 for all partitions. All other priors were let to defaults. Four independent analyses were performed, with Bayesian MCMC running for 20 million generations and sampling every 1000th generation. The results were inspected in Tracer 1.5, and 2 million, 2.1 million, 2 million and 2 million generations were discarded from each run as ‘burn-in’, respectively. Thereafter, the remaining results of all analyses were combined together using LogCombiner v1.6.2. The final tree was constructed using TreeAnnotator v1.6.2 in the High Performance Computing Center of University of Tartu and visualized in FigTree v1.4.0.

*Larentiinae*

Analysis focussed on the subfamily Larentiinae comprised a total of 108 species. For phylogenetic reconstruction, we first determined the best partitioning scheme from three competing scenarios. For the first scenario, each gene was defined as a separate partition; for the second scenario, each codon position (1^st^, 2^nd^ and 3^rd^) of each gene was defined as a separate partition; for the third scenario, the data were divided between 50 subsets (bins) according to their evolutionary rates using Tiger v 1.02. To choose between these scenarios, a ML tree following GTR+I+G model was first constructed using Mega6. This tree was used as user_tree_topology in PartitionFinder 1.1.1 to find the best partitioning scheme for all three competing scenarios. Bayesian Information Criterion (BIC) values of the best scheme for each scenario were compared and the bin-based treatment was selected for further calculations.

According to the suggestion by PartitionFinder, nucleotide data were divided into four partitions, each comprising one or more bins. GTR+I+G model was implemented on one, and GTR+G model on three partitions according to suggestions by PartitionFinder. A relaxed molecular clock allowing branch lengths to vary according to uncorrelated lognormal distribution was used for each partition. To calibrate the ultrametric tree, four calibration points were defined according to Wahlberg et al. 2013: root of Geometridae (age 71.5 million years, with uniform distribution between 60.9 and 82.6 million years), split between Archiearinae and Geometrinae+Ennominae lineage (age 60.5 million years, with uniform distribution between 49 and 71.5 million years), split between Ennominae and Geometrinae (age 46.1 million years, with uniform distribution between 35.8 and 56.2 million years), and split between Sterrhinae and Larentiinae (age 56 million years, with uniform distribution between 42.3 and 69.5 million years). The tree prior was set to the Birth–Death process whereas the ucld.mean parameter was given a uniform prior between 0 and 1 for all partitions. All other priors were let to defaults. Five independent analyses were performed, with Bayesian MCMC running for 20 million generations and sampling every 1000th generation. The results were inspected in Tracer 1.5, and 1 million, 6.5 million, 16 million, 10 million and 8.7 million generations were discarded from each run as ‘burn-in’, respectively. Thereafter, the remaining results of all analyses were combined together using LogCombiner v1.6.2. The final tree was constructed using TreeAnnotator v1.6.2 in the High Performance Computing Center of University of Tartu and visualized in FigTree v1.4.0.

*Sterrhinae*

Analysis focussed on the subfamily Sterrhinae comprised a total of 48 species. For phylogenetic reconstruction, we first determined the best partitioning scheme from three competing scenarios. For the first scenario, each gene was defined as a separate partition; for the second scenario, each codon position (1^st^, 2^nd^ and 3^rd^) of each gene was defined as a separate partition; for the third scenario, the data were divided between 50 subsets (bins) according to their evolutionary rates using Tiger v 1.02. To choose between these scenarios, a ML tree following GTR+I+G model was first constructed using Mega6. This tree was used as user_tree_topology in PartitionFinder 1.1.1 to find the best partitioning scheme for all three competing scenarios. Bayesian Information Criterion (BIC) values of the best scheme for each scenario were compared and the bin-based treatment was selected for further calculations.

According to the suggestion by PartitionFinder, nucleotide data were divided into four partitions, each comprising one or more bins. HKY+G and GTR+G models were both implemented on one, and GTR model on two partitions according to suggestions by PartitionFinder. A relaxed molecular clock allowing branch lengths to vary according to uncorrelated lognormal distribution was used for each partition. To calibrate the ultrametric tree, four calibration points were defined according to Wahlberg et al. 2013: root of Geometridae (age 71.5 million years, with uniform distribution between 60.9 and 82.6 million years), split between Archiearinae and Geometrinae+Ennominae lineage (age 60.5 million years, with uniform distribution between 49 and 71.5 million years), split between Ennominae and Geometrinae (age 46.1 million years, with uniform distribution between 35.8 and 56.2 million years), and split between Sterrhinae and Larentiinae (age 56 million years, with uniform distribution between 42.3 and 69.5 million years). An undated calibration point comprising the tribe Scopulini was also defined to stabilize the analysis. The tree prior was set to the Birth–Death process whereas the ucld.mean parameter was given a uniform prior between 0 and 1 for all partitions. All other priors were let to defaults. Three independent analyses were performed, with Bayesian MCMC running for 20 million generations and sampling every 1000th generation. The results were inspected in Tracer 1.5, and 1 million generations were discarded from each run as ‘burn-in’. Thereafter, the remaining results of all analyses were combined together using LogCombiner v1.6.2. The final tree was constructed using TreeAnnotator v1.6.2 in the High Performance Computing Center of University of Tartu and visualized in FigTree v1.4.0.

Second step: Construction of the final ultrametric tree

The three ultrametric trees which were obtained as a result of processes described in the previous section were concatenated into a single 366-species ultrametric tree. Initially, the consensus.edges function from the R (R Core Team 2012) package phytools was used to create a consensus of the subfamily-level sections of each of the three partitions to obtain lower-level consensus branch lengths between subfamilies (their estimates are slightly different in each partition). Then, the corresponding well-sampled species-level trees for each subfamily were grafted into their relevant places on the tree. The reason this process required two stages is because the consensus.edges function only handles fully-overlapping trees.

**References**

Cummins CA, McInerney JO (2011) A method for inferring the rate of evolution of homologous characters that can potentiallyimprove phylogenetic inference, resolve deep divergence and correct systematic biases. Syst Biol 60:833–844.

Drummond AJ, Ho SYW, Phillips MJ, Rambaut A (2006) Relaxed phylogenetics and dating with confidence. PLoS Biol 4:699–710.

Drummond AJ, Suchard MA, Xie D, Rambaut A (2012) Bayesian phylogenetics with BEAUti and the BEAST 1.7. Mol. Biol. Evol. 29: 1969-1973.

Gernhard T (2008) The conditioned reconstructed process. J Theor Biol 253:769-778

Hall, T.A. 1999. BioEdit: a user-friendly biological sequence alignment editor and analysis program for Windows 95/98/NT. Nucl. Acid S. 41: 95-98.

Hausmann, A., Haszprunar, G., Hebert, P.D.N. 2011. DNA barcoding the geometrid fauna of Bavaria (Lepidoptera): successes, surprises, and questions. PLoS ONE 6: e17134.

Holm, S., Davis, R.B., Javoiš, J., Õunap, E., Kaasik, A., Molleman, F. and Tammaru, T. 2016. A comparative perspective on longevity: the effect of body size dominates over ecology in moths. J. Evol. Biol., 29: 2422–2435.

Holm, S., Javoiš, J., Õunap, E., Davis, R., Kaasik, A., Molleman, F., Tasane, T & Tammaru, T. 2018. Reproductive behaviour indicates specificity in resource use: phylogenetic examples from temperate and tropical insects. Oikos 127: 1113-1124.

Huemer, P., Mutanen, M., Sefc, K.M. and Hebert, P.D. 2014. Testing DNA barcode performance in 1000 species of European Lepidoptera: large geographic distances have small genetic impacts. PLoS ONE 9: e115774.

Lanfear R, Calcott B, Ho SYW, Guindon S (2012) PartitionFinder: combined selection of partitioning schemes and substitution models for phylogenetic analyses. Mol Biol Evol 29:1695–1701.

Miller, M.A., Pfeiffer, W. & Schwartz, T. 2010 Creating the CIPRES Science Gateway for inference of large phylogenetic trees. Proceedings of the Gateway Computing Environments Workshop (GCE). New Orleans, Louisiana, 14 November 2010, pp. 1-8.

Mutanen, M., Wahlberg, N., Kaila, L. 2010. Comprehensive gene and taxon coverage elucidates radiation patterns in moths and butterflies. P. Roy. Soc. B-Biol. Sci. 277: 2839-48.

Õunap, E., Javois, J., Viidalepp, J., Tammaru, T. 2011. Phylogenetic relationships of selected European Ennominae (Lepidoptera: Geometridae). Eur. J. Entomol. 108: 267–273.

Õunap, E., Viidalepp, J., Saarma, U. 2008. Systematic position of Lythriini revised: transferred from Larentiinae to Sterrhinae (Lepidoptera, Geometridae). Zool. Scr. 37: 405–413.

Õunap, E., Viidalepp, J. & Truuverk, A. 2016. Phylogeny of the subfamily Larentiinae (Lepidoptera: Geometridae): integrating molecular data and traditional classifications. Syst. Entomol. 41: 824-843.

R CORE TEAM 2012. R: A language and environment for statistical computing. R Foundation for Statistical Computing, Vienna, Austria.

Revell, L. J. (2012), phytools: an R package for phylogenetic comparative biology (and other things). Methods in Ecology and Evolution, 3: 217–223.

Sihvonen, P., Mutanen, M., Kaila, L., Brehm, G., Hausmann, A., Staude, H.S. 2011. Comprehensive molecular sampling yields a robust phylogeny for geometrid moths (Lepidoptera: Geometridae). PLoS ONE 6: e20356.

Snäll, N., Tammaru, T., Wahlberg, N., Viidalepp, J., Ruohomäki, K., Savontaus, M.-L., Huoponen, K. 2007. Phylogenetic relationships of the tribe Operophterini (Lepidoptera, Geometridae): a case study of the evolution of female flightlessness. Biol. J. Linn. Soc. 92: 241–252.

Strutzenberger, P., Brehm, G., Bodner, F., Fiedler, K. 2010. Molecular phylogeny of *Eois* (Lepidoptera, Geometridae): evolution of wing patterns and host plant use in a species-rich group of Neotropical moths. Zool. Scr. 39: 603–620.

Tammaru, T., Johansson, N. R., Õunap, E. & Davis, R.B. 2018. Day-flying moths are smaller: evidence for ecological costs of being large. Journal of Evolutionary Biology 31: 1400-1404.

Tamura K, Stecher G, Peterson D, Filipski A, Kumar S (2013) MEGA6: Molecular Evolutionary Genetics Analysis Version 6.0. Mol Biol Evol 30: 2725–2729.

Thompson, J.D., Higgins, D.G., Gibson, T.J. 1994. ClustalW: improving the sensitivity of progressive multiple sequence alignment through sequence weighting, position specific gap penalties and weight matrix choice. Nucleic. Acids. Res. 22: 4673-4680.

Viidalepp, J., Tammaru, T., Snäll, N., Ruohomäki, K., Wahlberg, N. 2007. *Cleorodes* Warren, 1894 does not belong in the tribe Boarmiini (Lepidoptera: Geometridae). Eur. J. Entomol. 104: 303–309.

Wahlberg, N., Braby, M. F., Brower, A. V. Z., de Jong, R., Lee, M.-M. Nylin, S., Pierce, N. E., Sperling, F. A. H., Vila, R., Warren, A. D., Zakharov, E. 2005. Synergistic effects of combining morphological and molecular data in resolving the phylogeny of butterflies and skippers. P. Roy. Soc. B-Biol. Sci. 272: 1577-1586.

Wahlberg, N., Snäll, N., Viidalepp, J., Ruohomäki, K., Tammaru, T. 2010. The evolution of female flightlessness among Ennominae of the Holarctic forest zone (Lepidoptera, Geometridae). Mol. Phylogenet. Evol. 55: 929–938.

Wahlberg N, Wheat CW, Carlos Peña (2013) Timing and patterns in the taxonomic diversification of Lepidoptera (butterflies and moths). PLoS One 8:e80875.

Zrzavá, M., Hladová, I., Dalíková, M., Šíchová, J., Õunap, E., Kubíčková, S. & Marec, F. 2018. Sex chromosomes of the iconic moth *Abraxas grossulariata* (Lepidoptera, Geometridae) and its congener *A. sylvata*. Genes 9: 279.
