# Supplementary figures and images for: No Indication of High Host-Plant Specificity in Afrotropical Geometrid Moths
Source: J Insect Sci. 2019 Apr 30;19(3):1. doi: 10.1093/jisesa/iez028 (PMC6490970; doi:10.1093/jisesa/iez028)

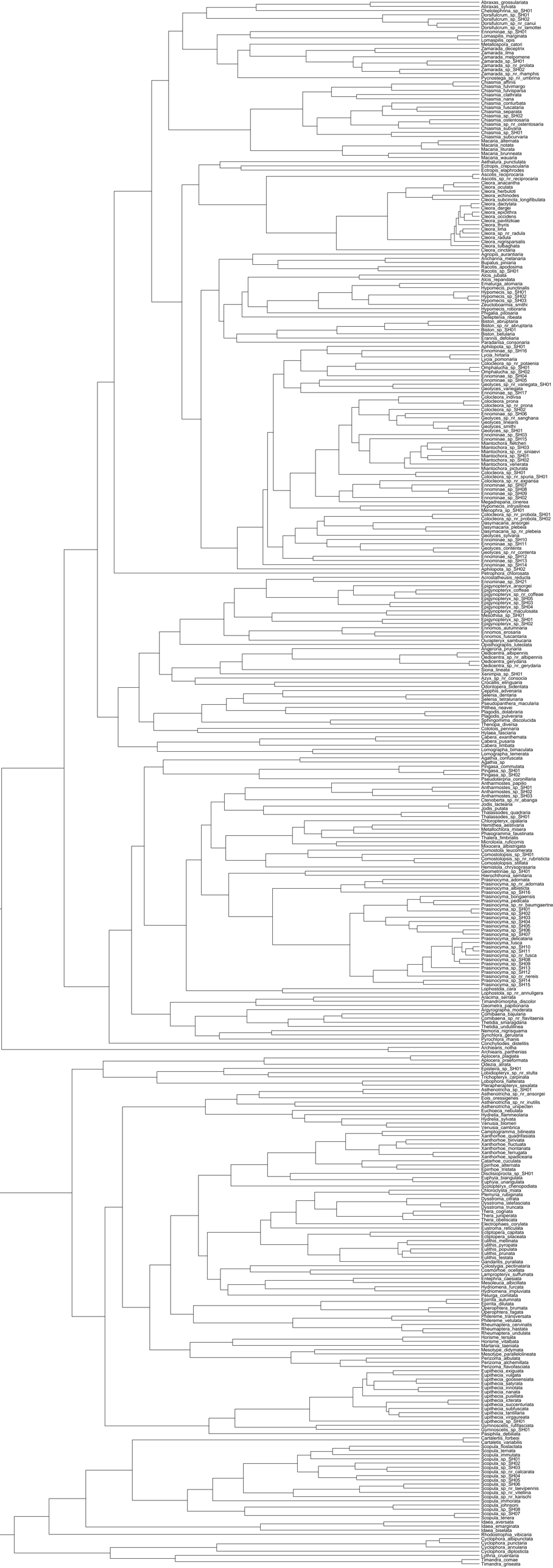

Supplement: iez028_suppl_Supplementary_Appendix_3 [file iez028_suppl_supplementary_appendix_3.pdf]
